# The course of pain hypersensitivity according to painDETECT in patients with rheumatoid arthritis initiating treatment: results from the prospective FRAME-cohort study

**DOI:** 10.1186/s13075-018-1581-4

**Published:** 2018-05-30

**Authors:** Signe Rifbjerg-Madsen, Anton Wulf Christensen, Mikael Boesen, Robin Christensen, Bente Danneskiold-Samsøe, Henning Bliddal, Lene Dreyer, Henning Locht, Kirstine Amris

**Affiliations:** 1 The Parker Institute, Copenhagen University Hospital, Bispebjerg and Frederiksberg, 2000 Frederiksberg, Copenhagen Denmark; 2 Department of Rheumatology, Copenhagen University Hospital, Bispebjerg and Frederiksberg, Copenhagen, Denmark; 3 Department of Rheumatology, Copenhagen University Hospital, Gentofte and Herlev, Hellerup, Denmark; 4 Department of Radiology, Copenhagen University Hospital, Bispebjerg and Frederiksberg, Copenhagen, Denmark

**Keywords:** Rheumatoid arthritis, Central sensitization, painDETECT questionnaire, Prognostics, Dynamic contrast-enhanced magnetic resonance imaging (DCE-MRI)

## Abstract

**Background:**

Evidence is emerging that pain in rheumatoid arthritis (RA) exists without underlying inflammation. Our objective was to evaluate the prognostic value of pain classification at treatment initiation using the painDETECT questionnaire (PDQ). Outcomes were change in DAS28-CRP and RAMRIS synovitis score.

**Methods:**

RA patients initiating a disease-modifying anti-rheumatic drug (DMARD) or initiating/ switching a biological agent were included. Follow-up time was 4 months. Clinical examination, imaging (MRI, dynamic contrast-enhanced MRI (DCE-MRI)), and patient-reported outcomes were undertaken. The PDQ was used to differentiate pain mechanisms. Mean change (95% CI) was calculated using ANCOVA. Multivariable regression models were used to determine a prognostic value.

**Results:**

A total of 102 patients were included; 75 were enrolled for MRI. Mean changes in baseline variables were greatest in the high PDQ classification group (> 18), while limited in the intermediate group (13–18). The 12 patients with high baseline PDQ score all changed pain classification group. No prognostic value of PDQ pain classification was found in relation to change of DAS28-CRP, RAMRIS score, or VAS pain. In the unadjusted model, RAMRIS score at baseline was associated with change in DAS28-CRP. The exploratory variables of DCE-MRI did not differ from other inflammatory variables.

**Conclusions:**

In RA patients a high PDQ score (non-nociceptive pain) at baseline was not associated with worse outcomes, in fact these patients had numerically greater improvement in DAS28-CRP. However, pain classification by PDQ was not independently associated with change in DAS28-CRP, RAMRIS score, or VAS pain in the prognostic models.

Furthermore, patients classified with a high baseline PDQ score changed pain classification group. Patients with unclear pain mechanism had reduced numerically treatment response.

**Trial registration:**

The study was approved by the Regional Ethics Committee of the Capital of Denmark April 18 2013; identification number H-3-2013-049.

**Electronic supplementary material:**

The online version of this article (10.1186/s13075-018-1581-4) contains supplementary material, which is available to authorized users.

## Background

Pain in rheumatoid arthritis (RA) has typically been regarded as nociceptive, that is, related to ongoing peripheral inflammation [1]. However, during the last decade, where focus has been on early diagnosis and aggressive treatment strategies in the treat-to-target regime [2], it has become clearer that in a subgroup of RA patients, pain can become an entity in its own right, probably elicited by, but not directly related to, ongoing inflammation [3]. A substantial proportion of RA patients in stable clinical remission continue to report moderate to severe pain levels [4] and studies have indicated that RA leads to widespread pain in 10–20% of patients [5]. Such observations have led to the contention that changes in the peripheral and central nervous system through processes of neural plasticity and central sensitization may play an important role [6]. As a rule, sensitization phenomena would be expected to extinguish as the tissue heals and inflammation subsides. However, a state of induced hypersensitivity of the pain system may persist in subsets of patients and lead to chronic pain states in which pain is no longer coupled to the presence of ongoing peripheral inflammation [7]. In such patients, persistent pain hypersensitivity may lead to continuous high reports of tender joints and poor global health; subcomponents of the commonly used composite disease activity score of 28 joints (DAS28-CRP) and thus overestimation of inflammatory activity. If the anti-inflammatory treatment is intensified on this background, little change in DAS28-CRP can be expected. Conversely, if inflammatory RA is left un- or not sufficiently treated it will lead to joint destruction and loss of function [8]. Identification of underlying pain mechanisms therefore has potential importance when prognosticating the effect of medical treatment on inflammation and pain.

The painDETECT questionnaire (PDQ) is a self-administered pain classification instrument originally developed to differentiate neuropathic (non-nociceptive) from non-neuropathic (nociceptive) pain [9]. It has been increasingly used in patients with osteoarthritis and fibromyalgia to assess clinical pain features indicative of central sensitization [10–12] and has recently been introduced in studies assessing pain mechanisms in patients with RA and spondyloarthritis [13–15].

Magnetic resonance imaging (MRI) is an objective and sensitive method to assess joint inflammation. The most common scoring system in the wrist and metacarpophalangeal (MCP) joints is the OMERACT (outcome measures in rheumatoid arthritis clinical trials) RA MRI scoring (RAMRIS) system [16]. Dynamic contrast-enhanced (DCE) MRI is a technique where the sequences are acquired rapidly and sequentially before and during contrast infusion. DCE-MRI has been shown to correlate better than conventional MRI with the histologic findings of synovitis [17, 18].

In this study, we hypothesized that a high PDQ score would serve as an indicator of central sensitization and thus a prognostic factor for a poorer treatment outcome (DAS28-CRP change) in patients with RA initiating or intensifying anti-inflammatory treatment. A possible statistical interaction between central sensitization (high PDQ score) and inflammatory load (baseline synovitis defined by hand MRI RAMRIS score) was considered as part of the hypotheses. In the exploratory part of the study, we hypothesized that DCE-MRI would capture change in inflammation and thus a possible relation to inflammatory pain mechanisms (low PDQ score) better than conventional MRI.

## Methods

### Design

The Frederiksberg Hospital’s Rheumatoid Arthritis, pain assessment and Medical Evaluation (FRAME)-cohort study was conducted according to a published protocol, which contained a detailed description of the methods and prespecified analysis [19, 20]. It was approved by the Regional Ethics Committee of the Capital of Denmark; identification number H-3-2013-049.

RA patients were recruited from departments and private clinics of rheumatology in the Copenhagen area and prospectively enrolled from March 2013 to September 2014. MRI was included in the examination program from May 2013. The examination program was conducted at Frederiksberg Hospital. Patients were assessed at treatment initiation (baseline) and after 4 months of treatment. Patients received routine care at the discretion of their rheumatologist during the trial period. Add-on of painkillers was allowed.

### Patients

To be eligible, patients had to fulfil either the 1987 [21] or 2010 ACR RA criteria [8] and be ≥18 years. Further, patients had to be scheduled for either (a) treatment initiation with any conventional synthetic disease-modifying antirheumatic drug (csDMARD) (patients who had not received treatment with csDMARD for at least 6 months including newly diagnosed/treatment-naïve patients) or (b) treatment initiation or change of any biologic DMARD (bDMARD).

Major exclusion criteria were intra-articular or intra-muscular glucocorticoids administered less than 3 weeks prior to baseline; treatment with oral corticosteroids at doses equivalent to more than 10 mg prednisolone/day within the 3 weeks prior to baseline; inability to pause antidepressants, anticonvulsants or other centrally acting analgesics; initiation of csDMARD therapy more than 3 weeks prior to the baseline visit (only patients initiating csDMARDs); treatment with bDMARD initiated more than 1 week prior to the baseline visit (only patients initiating a bDMARD). Patients who had contraindications for MRI were excluded from the MRI arm of the study. Furthermore, patients with increased risk of neuropathic pain conditions (e.g. diabetes) were excluded due to the potential to confound the pain assessment.

### Variables and outcome measures

The patients underwent an examination program at baseline and follow-up, collecting information on demographics and medication and from patient-reported outcomes (PROs). Clinical examination including joint count and tender point examination conducted by the same assessor at both time points, imaging (MRI and DCE- MRI), and standard blood samples (CRP, immunoglobulin M-rheumatoid factor [IgM-RF], anti-cyclic citrullinated peptide [anti-CCP]) were also performed.

The following PROs were collected from each patient; the PDQ, the Stanford health assessment questionnaire disability index (HAQ-DI), the 36-item short form health survey (SF-36), generalized anxiety disorder assessment (GAD-10) and major depression inventory (MDI).

The PDQ is a patient-administered pain classification tool that was developed in a population of patients with various pain conditions. It has been further validated for describing pain phenotypical features in patients with inflammatory arthritis by our group [22] and is validated for use on touch screen [9, 23]. It comprises items on pain intensity (three numeric rating scales not included in the total score), pain course patterns, pain radiation (from a pain drawing) and seven somatosensory signs and symptoms (rated on a six-category Likert scale). According to a validated algorithm, patients were assigned to one of three pain classification-groups based on a score between − 1 and 38: > 18 likely neuropathic pain, 13–18 unclear pain mechanism or < 13 likely non-neuropathic pain [9]. Several studies have used it as indicator of non-nociceptive or central pain mechanisms [10–12].

HAQ-DI is a measure of limitation of activities of daily living used for patients with RA. It assesses the patient’s ability to carry out everyday tasks. It includes a visual analogue scale (VAS) evaluation of pain, fatigue and global health (GH) [24].The SF-36 assesses eight domains concerning general health, which can be summarized into a physical (PCS) and mental (MCS) component summary score. In this study the Danish version of SF-36, which uses a 4-week recall period was applied [25]. The GAD-10 is a ten-item instrument developed from the Hamilton six-item anxiety scale. It measures generalized anxiety by scoring the total sum of the items [26]. The MDI is a questionnaire based on self-reported mood symptoms. It holds the ability to generate a DSM-IV and International Classification of Diseases (ICD)-10 diagnoses of major (moderate to severe) depression and to rate the severity of symptoms [27].

As standard research procedure a target hand was chosen for MRI to reflect the general level of joint inflammation. The most painful hand as reported by the patient was chosen, or, in case of no difference in pain level, the dominant hand. The examination was carried out in a 3 T Siemens Verio^®^ MR scanner according to a published scanning procedure [20]. Conventional coronal and axial STIR and 3D coronal T1w GRE VIBE pre- and post- contrast images were used for RAMRIS scoring. The wrist and MCP joints 2–5 were assessed according to the OMERACT RAMRIS [16, 28] and were scored for synovitis (0–3; total score 0–21) and bone marrow edema (BMO) (0–3; total score 0–58). Based on previous reports on smallest detectable difference, it was decided that the RAMRIS synovitis score had to alter by more than 1 unit to be considered a significant change [29]. All images were assessed blinded and paired by the same senior radiologist (MB).

For the explorative DCE-MRI analyses the software DYNAMIKA enterprise version 3.2.6 (http://www.ia-grp.com) was used according to a published procedure [30]. Only joints with MRI signs of inflammation (“focus joints”) were included. All images were analyzed paired by the same physician (SRM). Regions of interest (ROIs) were drawn on all slices where sign of inflammation was present and collapsed into one volume of interest (VOI) for each focus joint; wrist and 2nd-5th MCP. It was decided to include tenosynovitis and capsulitis as ‘signs of inflammation’ in the analyses. Joints with no signal were assigned a score of 0. Nvoxel, IRExNvoxel, MExNvoxel and IRExME were chosen as outcome measures [17, 31–35]. The number of enhancing voxels (Nvoxel) was multiplied by the volume of each voxel in milliliter (ml) to adjust for different image sizes. The initial rate of enhancement (IRE) and maximum enhancement (ME) represent the degree of perfusion; the IRE reflects the initial rate of enhancement of the time intensity curve. ME represents the equilibrium state of the curve and reflects the amount of contrast passing into the ROI. The composite outcome measures IRExNvoxel and MExNvoxel reflect both the volume and degree of perfusion, whereas IRExME characterizes the perfusion profile of the voxels derived from the time-intensity curves.

### Statistical analysis

SAS software (version 9.4, SAS Enterprise Guide 7.1, SAS Institute Inc., Cary, NC, USA) was used for all statistical analyses. PROC UNIVARIATE statement was used to summarize the data and for visual inspection of normality. Means (with standard deviations [SDs]) or medians (with interquartile ranges [IQRs]) were reported and compared by *t* test or Kruskal-Wallis (Wilcoxon) test, respectively. Delta changes were adjusted for baseline value and compared using analysis of covariance (ANCOVA). All analyses were carried out according to the intention-to-treat principle, i.e. missing data at follow-up was imputed from baseline (baseline observation carried forward). A two-sided *p* value less than 0.05 was regarded as being statistically significant.

Prior to executing the FRAME-cohort study, a power calculation was performed based on the assumption that it was feasible to include 100 RA patients during a study period of 1½ years [20], as no data for sample size calculation was available. Anticipating a common SD of 1.5 and the correlation between pre- and post-scores being *r* = 0.3 for a paired *t* test with a significance level of 0.05, a sample of a 100 pairs has a power of 80% (0.797) to detect a mean change of 0.5 DAS28-CRP units. A patient population, who can expect a change of this magnitude in their disease activity, is a reasonable cohort in which to study prognostic factors of treatment response. However, this number was not reached for the MRI subsample.

The prognostic value of the PDQ score, RAMRIS score, and their interaction at baseline in relation to change of DAS28-CRP was examined by multivariable regression models using the SAS PROC GLM. As the interpretation of PDQ by nature is trichotomous, the results were expressed as least squares means per category. According to the protocol, the model was adjusted for the following prespecified confounders: age (years), sex (male/female), disease duration (month), disease activity (DAS28-CRP at baseline), group (csDMARD/bDMARD), antiCCP-positive (yes/no) and concomitant prednisolone (yes/no). Subsequently, in the fully adjusted model covariates (i.e. possible confounders) that did not contribute to the model were removed; age, antiCCP-positive (yes/no) and concomitant prednisolone (yes/no).

Secondary outcomes were change in RAMRIS synovitis score and VAS pain. Post hoc, to ensure robustness of results, a sensitivity analysis including baseline VAS pain as a confounder in the adjusted analysis of change in VAS pain was performed.

On an exploratory basis the DCE-MRI variables IRExNvoxel (in ml) or MExNvoxel (in ml) for wrist were applied in the primary model examining DAS28-CRP and VAS pain change, replacing the RAMRIS synovitis score. For SRM, inter- and intra-reader agreements, intraclass correlation coefficients (ICC) (absolute agreement) for the four predefined DCE-MRI variables were tested beforehand on data from ten patients using SPSS software. Wrist and MCP joints were tested separately.

## Results

**Fig. 1:**
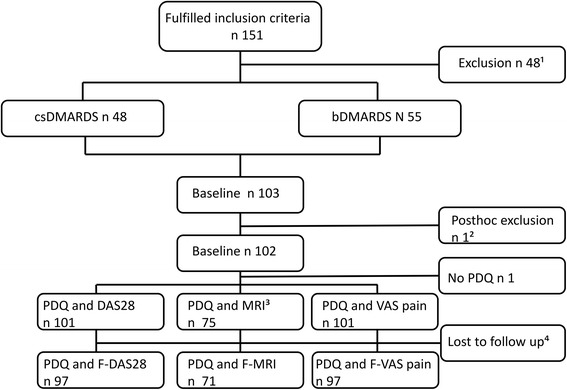
Flowchart of participants. 1: Refrained from participation (*n* = 18), comorbidity with risk of neuropathic pain (*n* = 10), unable to pause antidepressants, anticonvulsants, or other centrally acting analgesics for 1 week (*n* = 7), initiated DMARD treatment > 3 weeks ago (*n* = 5), received more than 10 mg prednisolone < 3 weeks ago (*n* = 4), other reasons (*n* = 4). 2: Im. Corticosteroid between screening and baseline assessment. 3: MRI not feasible or patients declined. 4: Four patients withdrew from the study; two from each treatment group, three from the MRI subgroup. A further one patient did not participate in the follow-up MRI

Figure 1 illustrates the flow of patients. In all, 151 patients fulfilled the inclusion criteria. Of these, 48 patients were excluded. In total, 103 patients received a baseline assessment; however two were excluded post hoc. In total, 101 patients completed the PDQ at baseline; 47 initiating csDMARD and 54 initiating bDMARD. Of these, 75 patients completed the PDQ and had an MRI scan performed at baseline and of these, 71 patients had an MRI scan performed at follow-up. This discrepancy was primarily caused by administrative delay resulting in no patients receiving MRI-scan during the first 2 months of the study period. Exclusion and reasons for dropout are further described in the figure text. Due to 3 patients not wishing to receive contrast, 72 patients with complete PDQ were included in the exploratory DCE-MRI study at baseline (not shown in Fig. 1). Of these72 patients, in all 65 completed the follow-up scan with contrast.

Baseline characteristics are described in Table 1 (some data has previously been published [36]). The distribution of patients across the PDQ classification groups were *n* = 66, *n* = 23, *n* = 12 for PDQ score < 13, 13–18, > 18, respectively. Statistically significant differences across the three PDQ classification groups were observed for tender joint count (TJC), tender point (TP) count, DAS28-CRP, physical function (HAQ-DI), VAS-fatigue, VAS-pain, VAS-GH, anxiety (GAD-10), depression (MDI), SF-36 PCS, and SF-36 MCS. A TP count ≥11 was found for higher proportions of patients with higher PDQ classification groups; however, this was not statistically significant. No differences were found across the groups for the biochemical and imaging variables.

**Table 1:** Baseline characteristics stratified by PDQ group

	PDQ score 7 (5-9)	PDQ score 16 (13-18)	PDQ score ≥ 19	*p* value
	(*n* = 66)	(*n* = 23)	(*n* = 12)	
PDQ score	7 (5–9)	16 (13–18)	21 (20–23)	<.0001
Female, n (%)	45 (68.2)	20 (87.0)	31 (91.7)	0.09
Age, years (SD)	56.1 (14.7)	54.6 (18.9)	47.4 (15.6)	0.22
Initiated csDMARD, n (%)	37 (78.7)	6 (12.8)	4 (8.5)	
Disease duration, months	15.5 (1–104)	53 (9–47)	34.5 (24–149.5)	0.12
Current smoker, n (%)	11 (16.9) ^a^	7 (30.4)	2 (16.7)	0.39
Corticosteroid usage, n (%)	11 (16.7)	2 (8.7)	3 (25.0)	0.42
28 Swollen joint count	2 (1–7)	5 (2–8)	2 (1–4)	0.09
28 Tender joint count	5 (3–10)	13 (8–16)	9.5 (6.5–15)	<.0001
Tender point count, 0–18	6 (4–14)	10 (7–16)	12 (6–14)	0.02
Tender point count ≥11, n (%)	20 (30.3)	11 (47.8)	7 (58.3)	0.09
DAS28 (SD)	4.2 (1.1)	5.0 (1.2)	4.8 (0.7)	0.007†
HAQ-DI, 0–3	0.75 (0.38–1.25)	1.13 (0.88–1.75)	1.63 (1.19–1.88)	0.0004
VAS-fatigue, mm	53.5 (27–71)	75 (52–89)	72.5 (60.5–87)	0.002
VAS-pain, mm	42 (24–60)	69 (50–82)	63 (45.5–80.5)	0.0003
VAS-global health, mm	56 (32–77)	73 (46–85)	78 (61.5–90)	0.03‡
GAD-10 score, 0–50	6 (3–10) ^a^	10 (6–17)	9 (9–12)	0.008
MDI score, 0–50	8 (4–12) ^a^	11 (6–25)	13.5 (9–19.5)	0.01
SF-36 PCS, 0–100	35 (29–42)	33 (26–37)	28 (24–32)	0.02‡
SF-36 MCS, 0–100	51 (40–57)	43 (31–51)	37 (31–49)	0.005
CRP, mg/mL	8 (3–15)	4 (0.5–19)	3 (0.8–8.5)	0.13
IgM-RF positive, n (%)	41 (62.1)	15 (65.2)	8 (66.7)	1.0
Anti-CCP positive, n (%)	46 (70)	15 (65)	7 (58)	0.72
RAMRIS hand synovitis	7 (5–10)	9.5 (7–11)	7 (6–9)	0.18
RAMRIS hand edema	5 (1–10)	9.5 (3–22)	5 (2–13)	0.12

Values are median (25th, 75th percentiles) unless specified otherwise

Unless specified otherwise significant *p* values reflect difference between PDQ score < 13 and 13–18 and ≥ 19, while there is no difference between PDQ score 13–18 and ≥ 19

^†^Only difference between PDQ score < 13 and 13–18

^‡^Only difference between PDQ score < 13 and ≥ 19

RAMRIS hand; wrist+MCP scores

One patient receiving MRI had no corresponding PDQ score. 26 patients did not receive MRI scan. Number of patients (n) for RAMRIS parameters: PDQ score < 13, *n* = 51; PDQ score 13–18, *n* = 18; PDQ score > 18, *n* = 6. (a) One missing observation. (b) Three missing observations

Changes from baseline stratified by PDQ group are presented in Table 2. Change in PDQ classification group is reported as classification consistency, i.e. number of patient that did not change classification group. Statistically significant differences in classification consistency were found for the low, intermediate, and high PDQ group; 97%, 83%, and 0%, respectively. For all clinical variables and PROs, a U-shaped curve change pattern was observed with greater change in the high and low PDQ classification group than in the intermediate. Remission at follow-up according to DAS28-CRP (< 2.6) was found in 41%, 22%, and 42%, respectively. The change in imaging variables did not display a distinct pattern. Statistically significant differences between the classification groups were found for delta change of CRP, DAS28-CRP, VAS GH, HAQ-DI, and SF36- MCS. However for CRP, no differences between PDQ score < 13 and > 18, and 13–18 and > 18 were found. For DA28-CRP and VAS GH no differences were found between PDQ score < 13 and PDQ score > 18. Regarding HAQ-DI and SF36-MCS there were no difference between PDQ score < 13 and 13–18. DCE-MRI variables are presented separately in Table 3. There were neither significant differences nor trends in the DCE-MRI variables across the groups; however, the variables IRExNvoxel (in ml) and MExNvoxels (in ml) for the wrist also displayed the U-shaped change pattern in line with the RAMRIS score (BME). Baseline characteristics and delta change by initiation group have previously been reported [36, 37].

**Table 2:** Mean change stratified by baseline PDQ group adjusted for baseline value

	PDQ score < 13	PDQ score 13–18	PDQ score ≥ 19	*p* value
	(*n* = 66)	(*n* = 23)	(*n* = 12)	
PDQ consistency, N (%)	64 (97)	19 (83)	0 (0)	< 0.001^†^
Δ PDQ score	−4.10 (−5.74;-2.45)	−2.20 (− 4.64;0.25)	−5.49(−9.97;-1.02)	0.08
Δ 28 SJC	−2.71 (− 3.26;-2.17)	− 2.27 (− 3.21;-1.34)	− 2.48(− 3.77;-1.19)	0.71
Δ 28 TJC	− 4.63(− 5.67;-3.60)	−4.36(− 5.14; − 1.58)	−5.57(−7.96;-3.18)	0.28
Δ CRP, mg/mL	−6.93 (− 8.54;-5.32)	−2.81 (− 5.54;-0.08)	−6.28 (− 10.10;- 2.47)	0.04a
Δ DAS28-CRP	− 1.47 (− 1.74; − 1.20)	−0.8(− 1.27;-0.34)	−1.62 (− 2.25;-1.00)	0.03b
Δ Tender point count	−2.70 (− 3.87;-1.57)	−1.56 (− 3.48; 0.36)	− 3.83 (− 6.46;-1.20)	0.35
Δ VAS-fatigue	−18.31(− 25.55;-13.06)	−8.19 (− 17.07; 0.68)	−22.20 (− 34.48;-9.91)	0.09
Δ VAS-pain	−23.27(− 28.21;-18.33)	−12.99(− 21.40;-4.57)	−26.86 (− 38.34;-15.38)	0.07
Δ VAS-global health	−26.20(− 31.15;-21.25)	−13.49 (− 21.83;-5.15)	−31.72 (− 43.44;-20.01)	0.01b
Δ HAQ-DI	− 0.34 (− 0.44;-0.24)	− 0.15 (− 0.32; 0.02)	−0.74 (− 0.98;-0.50)	< 0.001c
Δ MDI-total	− 3.04(− 4.39;-1.68)	−2.54 (− 4.83;-0.25)	−3.68(− 6.80;-0.55)	0.84
Δ GAD10-total	−2.24 (− 3.18;-1.30)	−1.56 (− 3.15; 0.02)	−2.10 (− 4.27; 0.07)	0.76
Δ SF36-MCS	5.5 (3.34; 7.60)	3.70 (0.09; 7.31)	11.38 (6.40;16.36)	0.04c
Δ SF36-PCS	6.47 (4.39; 8.56)	4.94 (1.43; 8.44)	8.96 (3.97; 13.94)	0.42
Δ RAMRIS H synovitis	−1.69 (− 2.32;-1.07)	− 1.81(− 2.90,-0.71)	−2.47 (− 4.28;-0.66)	0.72
Δ RAMRIS H edema	−2.17 (− 4.00;-0.34)	− 0.57(− 3.80;2.66)	− 3.46 (− 8.72;1.81)	0.58

Values are means (95% CI). Negative numbers indicate improvement. ANCOVA (BOCF) was used for the analyses unless otherwise indicated. †Chi-square test

RAMRIS *H* hand (W + M). *W* wrist. *M* MCP joints

(n) for RAMRIS parameters: PDQ score < 13; *n* = 51, PDQ score 13–18; *n* = 18, PDQ score > 18; *n* = 6. (a) No difference between PDQ score < 13 and > 18, and 13–18 and > 18. (b) No difference between PDQ score < 13 and PDQ score > 18. (c) No difference between PDQ score < 13 and 13–18

**Table 3:** Baseline values and mean changes (adjusted for baseline value) for the exploratory DCE-MRI variables

	PDQ score < 13	PDQ score 13–18	PDQ score ≥ 19	*p* value
Baseline values				
Nvoxel in ml wrist	13.17 (5.53–29.40)	14.78(7.22–25.84)	14.21(8.28–22.70)	0.93
Nvoxel in ml MCP	1.65 (0.47–6.57)	3.12(0.80–9.22)	1.69(0.00–3.91)	0.56
IRE x Nvoxel in ml wrist	0.15 (0.05–0.67)	0.25(0.04–0.67)	0.19(0.06–0.56)	0.91
IRE x Nvoxel in ml MCP	0.02 (0.002–0.15)	0.06(0.003–0.24)	0.006(0.00–0.07)	0.31
ME x Nvoxel in ml wrist	21.43(9.76–62.29)	26.65(10.09–56.40)	26.73(14.72–42.67)	0.92
ME x Nvoxel in ml MCP	2.30 (0.67–14.23)	6.70(0.91–21.34)	2.46(0.00–7.42)	0.50
IRE x ME wrist	0.02 (0.01–0.05)	0.03(0.01–0.06)	0.02(0.01–0.05)	0.87
IRE x ME MCP	0.03 (0.01–0.09)	0.04(0.01–0.12)	0.01(0.00–0.05)	0.21
Mean changes				
Δ Nvoxel in ml wrist	− 2.22 (− 4.84;0.41)	− 2.47(− 6.93;1.99)	− 2.87(− 10.37;4.63)	0.98
Δ Nvoxel in ml MCP	− 1.99 (− 3.02;-0.96)	− 1.72(− 3.47;0.03)	−0.79 (− 3.74;2.17)	0.74
Δ IRE x Nvoxel in ml wrist	−0.18(− 0.28;-0.07)	−0.11(− 0.29;0.07)	−0.17(− 0.46;0.13)	0.80
Δ IRE x Nvoxel in ml MCP	−0.07 (− 0.11;-0.03)	−0.06(− 0.13;0.003)	−0.06(− 0.17;0.06)	0.99
Δ ME x Nvoxel in ml wrist	−7.72 (− 14.69;-0.76)	−5.55(− 17.37;6.27)	−7.90(− 27.80;12.00)	0.95
Δ ME x Nvoxel in ml MCP	−5.17(− 7.42;-2.92)	− 4.82(− 8.64;-1.00)	−3.12(− 9.58;3.33)	0.84
Δ IRE x ME wrist	−0.01 (− 0.02;− 0.01)	−0.01(− 0.02;0.003)	-0.01(− 0.03;0.01)	0.81
Δ IRE x ME MCP	− 0.03(− 0.04;-0.02)	−0.03(− 0.06;-0.01)	−0.03(− 0.07;0.01)	0.96

Baseline values are medians (25th, 75th percentiles)

PDQ score < 13, *n* = 49; PDQ score 13–18, *n* = 17; PDQ score > 18: *n* = 6

In the multivariable regression models (Table 4) change in the predefined outcome variables was expressed as least square means. No interaction was found between PDQ classification group and RAMRIS synovitis neither in the protocolized analysis, nor between PDQ classification group and the DCE-MRI variables (IRExNvoxel (ml), MExNvoxel (ml)) in the exploratory analysis. All interaction statements were therefore left out of the models. No prognostic value of PDQ classification was found for any of the outcomes in any of the models, sensitivity analysis included (*p* = 0.44) (data for DCE-MRI is presented as additional material, Additional file 1).

**Table 4:** Multivariable regression models examining change expressed as least squares means (95% CI)

Protocolized models	PDQ score < 13	PDQ score 13–18	PDQ score > 18	*p* value
Unadjusted model				
ΔDAS28	−1.30(− 1.64;-0.97)	−1.17(− 1.74;-0.60)	− 2.08(− 3.06;-1.10)	0.27
ΔRAMRIS Synovitis	−1.68(− 2.30;-1.06)	−1.75(− 2.80;-0.70)	−2.46(− 4.25;-0.66)	0.72
ΔVAS pain	−17.57(− 24.13;-11.02)	− 25.45(− 36.56;-14.33)	−38.13(− 57.15;-19.11)	0.09
Adjusted				
ΔDAS28	−1.49 (− 1.81;-1.18)	−1.16(− 1.70;-0.61)	−2.06(− 2.94;-1.17)	0.17
ΔRAMRIS Synovitis	−1.78(− 2.44;-1.26)	−1.72(− 2.86;-0.57)	−2.48(− 4.34;-0.63)	0.74
ΔVAS pain	− 19.36(− 25.89;-12.83)	−24.71(− 36.07;-13.35)	−38.04(− 56.50;-19.56)	0.15

No interaction was found in any of the models. The protocolized unadjusted models included the baseline PDQ classification groups as a trichotomous variable and RAMRIS synovitis score for the hand. The adjusted models further included female sex, disease duration, initiation group, and DAS28-CRP as covariates

Protocolized models, n: PDQ score < 13; *n* = 51, PDQ score 13–18; *n* = 18, PDQ score > 18; *n* = 6

Baseline DAS28 was found to be statistically significantly associated with change in all outcomes in both adjusted models; DAS28, RAMRIS, and VAS pain (*p* < 0.01), sensitivity analysis excluded. In this analysis, baseline VAS pain was found to be significantly associated with change in VAS pain (*p* < 0.001). Baseline RAMRIS synovitis score was found to be statistically significantly positively associated with DAS28-CRP change in the unadjusted model (*p* = 0.01) and of RAMRIS change in both the unadjusted and the adjusted model (*p* ≤ 0.02).

In the exploratory DCE-MRI study inter and intra-reader reliability showed good to excellent agreement (data is shown in Additional file 2).

## Discussion

To our knowledge, this is the first study to evaluate the prognostic value of pain classification by the PDQ score in relation to change in DAS28-CRP, VAS pain, and RAMRIS score in RA patients initiating or escalating anti-inflammatory treatment. It was hypothesized that patients in the high PDQ classification group (score > 18) would display constant high TJC and VAS GH as features of persistent pain hypersensitivity (central sensitization) and thus gain little or no change of DAS28-CRP following treatment initiation. In contrast however, this was not confirmed in the multiple regression analysis, we found that these patients (*n* = 12) experienced the greatest numerical change in DAS28-CRP, self-reported disease severity measures, and objective inflammatory parameters, including MRI (Table 2). This result was seen despite the fact that baseline inflammatory parameters were the same across the three PDQ classification groups, and that other baseline characteristics of the high PDQ classification group clinically could indicate presence of central sensitization (high frequency of female sex, a high number of tender joints and tender points, poor mental well-being, a high disability index, and high VAS scores).

In contrast to the patients in the low and intermediate PDQ classification -group, all patients in the high PDQ classification group changed pain classification following treatment initiation or escalation. These findings could indicate that in these particular patients, a high PDQ score might not have marked central pain mechanisms uncoupled to ongoing inflammation, but could have reflected reversible inflammatory driven pain hypersensitivity, i.e. normal neuroplasticity. However, regression toward the mean needs to be taken into account. Interestingly, the observed numerically changes of the variables were smallest in the intermediate PDQ group (score 13–18), total hand RAMRIS synovitis score excluded. Thus, patients with an unclear pain mechanistic background had the poorest response to medical treatment also reflected in the U-shaped DAS28 remission pattern across the PDQ classification groups, indicating that the uncoupling of pain mechanism from present inflammation could be found in this group.

In the multivariable regression analyses, the PDQ did not have prognostic value in relation to change of DAS28-CRP, RAMRIS score, or VAS pain. No interaction between PDQ score and baseline RAMRIS synovitis score was found in any of the regression analyses including the exploratory DCE-MRI analyses indicating no relation between ongoing inflammation and pain phenotype (pain classification group). However, in the light of the relatively small sub-sample of patients with a high PDQ score at baseline, it does not seem reasonable to reject any prognostic value of the PDQ at this point.

In contrast, DAS28-CRP at baseline was a significant predictor (*p* < 0.01) of change in the dependent variable (DAS28-CRP, RAMRIS synovitis score, VAS pain) in all adjusted models. Furthermore, RAMRIS synovitis predicted DAS28-CRP change in the unadjusted model (*p* = 0.01), and RAMRIS synovitis change both in the unadjusted and the adjusted model (*p* ≤ 0.02). The impact of RAMRIS synovitis score on clinical change is not well documented [38, 39]. Although our group previously has found correlations between RAMRIS synovitis score and DAS28-CRP in another cohort of RA patients (in review), this may be the first study to indicate a possible prognostic value of RAMRIS synovitis score in relation to DAS28-CRP change within 4 months. The clinical consistency and relevance of this finding needs further investigation.

Overall the DCE-MRI analyses did not add further information to conventional MRI.

The unexpected behavior of the PDQ may lead to speculations about whether the presents finding are due to the PDQ not being valid in this population. However, the PDQ has, in at least three cross-sectional studies [13, 14, 36], indicated involvement of non-nociceptive pain mechanisms in subgroups of RA patients. Although we failed to demonstrate a prognostic value of the PDQ, the implications may still be that in patients with clear indication of non-nociceptive pain mechanisms treatment strategies should involve management of chronic pain, including treatment with classes of medication that target central pain mechanisms [6, 7, 40–43], and not only focus on medical treatment of the underlying disease.

The main strength of this study was the prospective design with a rigorous protocol and a prespecified analysis plan. Furthermore, the sample of RA patients was heterogeneous including patients from outpatient clinics and private rheumatologists in the Copenhagen area with a broad spectrum of disease severity. Thus, on one hand this heterogeneity gave the results potential for generalizable interpretation, on the other hand, it was the main limitation in relation to pain profiling, i.e. the constitution of the sample with notably the subgroup of patients with a high baseline PDQ score was small (*n* = 12).

Inflammation was not only assessed clinically but also by MRI of one hand to ensure objectivity. Assessing one hand as a target joint area to reflect general inflammation is a common procedure but can be a limitation to the regression analysis as it involves a risk of misestimating the inflammatory load especially in patients with a non-typical RA presentation. However, it has not yet been clarified how many joints that should be included in a prognostic image analysis to reflect overall inflammatory load.

The study was limited by the framework of the PDQ; the original development for classification of neuropathic pain among patients with various chronic pain conditions, the algorithm for cutoff points also being validated in this sample and the lack of a clinical ‘gold standard’ assessing augmented central pain mechanisms, against which the PDQ preferably should be tested. However, patients with augmented central pain mechanisms express the same pain features as patients with neuropathic pain, though in a generalized pattern [12, 44–46]. In our opinion this sensitive but not specific feature of the questionnaire, association to tender point count and sign of central sensitization in quantitative sensory testing and functional MRI findings within other chronic pain conditions vouch for the use of the PDQ as indicator of augmented central pain processing [10–12, 47–49].

The relatively short follow-up time of 4 months is a limitation that may have reduced the treatment response for some patients. Further, the baseline visits including MRI could be up to 3 weeks after starting therapy for patients initiating a csDMARD and up to 1 week for patients initiating a bDMARD, which also may have reduced the observed treatment change from baseline to follow-up, particularly on DCE-MRI that is known to be very sensitive to fast inflammatory changes [31, 35, 50, 51]. Finally, a direct pain modulating effect of anti-tumor necrosis factor (TNF)-alpha treatment is indicated by the literature [52], and it could therefore be speculated that in some patients treated with anti-TNF blocker the allowed delay in baseline assessment may have influenced nociceptive signaling and thereby the study findings.

## Conclusions

In this study, higher numerically changes of inflammatory and patient-reported outcomes, and change of PDQ classification-group at follow-up were observed in the group of patients with a high PDQ score at baseline, which may indicate that inflammatory pain can lead to pain hypersensitivity of a reversible character. Interestingly, overall reduced change in variables was found in patients in the intermediate PDQ classification group with unclear pain mechanism indicating irreversible pain mechanisms. In contrast to our hypothesis, a high PDQ score had no prognostic value in relation to treatment outcome specified as change in DAS28-CRP, RAMRIS score, DCE-MRI, or VAS pain 4 months after initiation or escalation of medical therapy, however, it was found that the RAMRIS synovitis score may have prognostic value in relation to DAS28-CRP response. Further large-scale studies are needed to clarify the prognostic value of the PDQ.

## Additional files

Additional file 1: Table showing inter- and intra-reader reliability as intraclass correlation coefficient (ICC) for the DCE-MRI variables. (DOCX 16 kb)

Additional file 2: Table of multivariable regression models including DCE-MRI variables examining change across PDQ categories expressed as least squares means (95% CI). (DOCX 16 kb)

## Abbreviations

ANCOVA: Analysis of covariance
Anti-CCP: Anti-cyclic citrullinated peptide
CRP: C-reactive protein
DAS28-CRP: Disease activity score 28 joints – C-reactive protein
DCE-MRI: Dynamic contrast- enhanced magnetic resonance imaging
DMARD: Disease-modifying antirheumatic drug
FM: Fibromyalgia
GAD-10: Generalized anxiety disorder assessment
GH: Global health
HAQ: Health-assessment questionnaire
IRE: Initial rate of enhancement
IQR: Interquartile range
MCP: Metacarpophalangeal
MCS: Mental component score
MDI: Major depression inventory
ME: Maximum enhancement
MRI: Magnetic resonance imaging
MTX: Methotrexate
Nvoxel: Number of enhancing voxel
PCS: Physical component score
PDQ: painDETECT questionnaire
PRO: Patient-reported outcome
RA: Rheumatoid arthritis
RAMRIS: Rheumatoid arthritis magnetic resonance imaging score
RF: Rheumatoid factor
ROI: Region of interest
SF-36: 36-item short form health survey
SJC: Swollen joint count
TJC: Tender joint count
TNF: Tumor necrosis factor
VAS: Visual analogue scale
