# Supplementary material for: The course of pain hypersensitivity according to painDETECT in patients with rheumatoid arthritis initiating treatment: results from the prospective FRAME-cohort study
Source: Arthritis Res Ther. 2018 May 30;20:105. doi: 10.1186/s13075-018-1581-4 (PMC5977471; doi:10.1186/s13075-018-1581-4)
Supplement: Supplementary file 1 — Table showing inter- and intra-reader reliability as intraclass correlation coefficient (ICC) for the DCE-MRI variables. (DOCX 16 kb) [file 13075_2018_1581_MOESM1_ESM.docx]

**ADDITIONAL FILE 1.**

Multivariable regression models including DCE-MRI variables examining change expressed as least squares means (95% CI).

| EXPLORATORY MODELS  UNADJUSTED MODEL | PDQ score <13 | PDQ score 13-18 | PDQ score >18 | P-value |
| --- | --- | --- | --- | --- |
| ΔDAS28 (IRExNvoxel(ml)) | -1.23(-1.58;-0.88) | -1.32(-1.91;-0.73) | -2.15(-3.14;-1.15) | 0.23 |
| ΔDAS28 (MExNvoxel(ml)) | -1.22(-1.57;-0.87) | -1.35(-1.94;-0.76) | -2.11(-3.11;-1.12) | 0.25 |
| ΔVAS pain (IRExNvoxel(ml)) | -17.70(-24.46;-10.95) | -26.22(-37.71;-14.72) | -38.15 (-57.47;-18.83) | 0.09 |
| ΔVAS pain (MExNvoxel(ml)) | -17.68(-24.43;-10.93) | -26.31(-37.76;-14.85) | -38.07(-57.35;-18.79) | 0.09 |
| ADJUSTED |  |  |  |  |
| ΔDAS28 (IRExNvoxel(ml)) | -1.46(-1.79;-1.14) | -1.23(-1.79;-0.67) | -2.05(-2.94;-1.15) | 0.25 |
| ΔDAS28 (MExNvoxel(ml)) | -1.46(-1.78;-1.14) | -1.24(-1.79;-0.68) | -2.04(-2.93;-1.14) | 0.27 |
| ΔVAS pain (IRExNvoxel(ml)) | -18.92(-25.70;-12.14) | -24.88(-36.67;-13.08) | -36.83(-55.76;-17.89) | 0.18 |
| ΔVAS pain (MExNvoxel(ml)) | -18.10(-25.83;-12.17) | -24.60(-36.46;-12.75) | -37.31(-56.34;-18.28) | 0.18 |

In these exploratory models the dynamic contrast enhanced (DCE) MRI variables wrist IRExNvoxels (ml) and wrist MExNvoxels (ml), respectively, replaced the hand RAMRIS score.

Exploratory models, n: PDQ-score <13; n = 49, PDQ-score 13-18; n = 17, PDQ-score >18; n = 6
