# Supplementary material for: The course of pain hypersensitivity according to painDETECT in patients with rheumatoid arthritis initiating treatment: results from the prospective FRAME-cohort study
Source: Arthritis Res Ther. 2018 May 30;20:105. doi: 10.1186/s13075-018-1581-4 (PMC5977471; doi:10.1186/s13075-018-1581-4)
Supplement: Supplementary file 2 — Table of multivariable regression models including DCE-MRI variables examining change across PDQ categories expressed as least squares means (95% CI). (DOCX 16 kb) [file 13075_2018_1581_MOESM2_ESM.docx]

**ADDITIONAL file 2.**

Table showing inter- and intra-reader reliability as intraclass correlation coefficient (ICC) for the DCE-MRI variables.

| Variables | Inter-reader ICC (95% CI)  MCP Wrist | | Intra-reader ICC (95%CI)  MCP Wrist | |
| --- | --- | --- | --- | --- |
| Nvoxel | 0.91 (0.66;0.98) | 0.89 (0.26;0.98) | 0.98 (0.93;0.99) | 0.97 (0.88;0.99) |
| IRExNvoxel | 0.86 (0.49;0.97) | 0.98 (0.87;0.99) | 0.97 (0.90;0.99) | 0.99 (0.95;0.99) |
| MExNvoxel | 0.92 (0.71;0.98) | 0.94 (0.53;0.99) | 0.99 (0.95;0.99) | 0.98 (0.93;0.99) |
| IRExME | 0.97 (0.90;0.99) | 0.99 (0.96;0.99) | 0.98 (0.93;0.99) | 0.97 (0.88;0.99) |
